# A preliminary study of the anti-κ myeloma antigen monoclonal antibody KappaMab (MDX-1097) in pretreated patients with κ-restricted multiple myeloma

**DOI:** 10.1038/s41408-019-0217-5

**Published:** 2019-07-31

**Authors:** Andrew Spencer, Patricia Walker, Parisa Asvadi, Douglas H. Campbell, Kate Reed, Ben R. Herbert, Edmond J. Breen, Michael C. Copeman, Rosanne D. Dunn

**Affiliations:** 1 Alfred Health-Monash University, Melbourne, VIC Australia; 2 Aurobindo Pharma Ltd, Hyderabad, AP India; 3 Minomic International Ltd, Sydney, NSW Australia; 4 Kolling Institute, Northern Sydney Local Health District, Sydney Medical School Northern, University of Sydney, St Leonards, NSW Australia; 5 Australian Proteome Analysis Facility, Macquarie University, Sydney, NSW Australia; 6 45 Baringa Road, Northbridge, NSW Australia; 7 HaemaLogiX Pty Ltd, Sydney, NSW Australia

**Keywords:** Cancer immunotherapy, Lymphoproliferative disorders, Myeloma, Cancer microenvironment

## To the Editor

Despite increased survival rates over the past two decades in patients with multiple myeloma (MM), incurability remains an ongoing clinical challenge^1^. MM is characterised by the expansion of malignant monoclonal plasma cells (PCs) within the bone marrow (BM), and production of excess monoclonal immunoglobulin (M protein) and/or isotype-restricted free light chains (FLC)^2,3^.

Novel MM therapies such as the proteasome inhibitors bortezomib and carfilzomib, as well as the immunomodulatory drugs (IMiDs®) such as thalidomide, lenalidomide and pomalidomide, have had a major impact on survival rates^4^. However, drug resistance is common, with inevitable disease relapse. More recently, monoclonal antibodies (mAbs) such as elotuzumab, daratumumab, and isatuximab, which target cell-surface markers expressed on myeloma cells and induce antibody-dependent cellular cytotoxicity (ADCC), have been introduced into treatment regimens. These mAbs have favourable safety profiles and are often more effective when used in combination with proteasome inhibitors and/or IMiDs®^5^.

Our mAb, MDX-1097 (also called KappaMab), is an IgG1k chimeric mAb that binds to a unique conformational epitope on the κ myeloma antigen (KMA), which is a membrane-bound form of κ light chain that is present on malignant B cells such as κ-restricted MM cells, some lymphoma cells including those associated with Waldenstroms macroglobulinemia, some tonsillar B cells and in vitro-activated B cells^6^. MDX-1097 binds to serum κ FLC (κFLC), albeit with lower affinity than to malignant MM cells, and importantly, KMA is not found on normal human leucocytes^6–8^. KMA is non-covalently associated with lipids in the plasma membrane and MDX-1097 binding to KMA may induce changes to the cell membrane, potentially activating immune cell signal transduction pathways^7^. Further, our in vitro studies demonstrated that MDX-1097 induces ADCC and this effect is augmented in the presence of lenalidomide^7^. Given that KMA is expressed on malignant B cells, we feel that this mAb has therapeutic potential for treating MM. Here we report the results of a first-in-human dose-escalation study to assess safety and pharmacokinetics/pharmacodynamics (PK/PD) of MDX-1097 in patients with κ-restricted MM (Clinical trial registry: www.anzctr.org.au; ACTRN12608000336381).

Patients had received ≥2 prior lines of therapy (between 2 and 11 antineoplastic lines; details are in Supplementary Table 1), achieved at least a prior minimal response and at the time of enrolment had persistent, stable disease. Patients on maintenance therapy (9 out of 12; 75%) continued these treatments during the study (Table 1). Single intravenous (IV) infusions of ascending MDX-1097 doses (0.3, 1.0, 3.0 and 10.0 mg/kg) were administered. Safety, PK/PD, exploratory biomarkers, and immunogenicity (see Supplementary Methods) were evaluated up to 135 days post infusion. The primary goal was to evaluate the safety profile of MDX-1097 at four dose levels. As patients only received a single infusion of MDX-1097, it was not anticipated that conventional responses would be observed.

**Table 1:** Patient baseline characteristics and adverse events after treatment with MDX-1097

	Dose of MDX-1097				
	0.3 mg/kg (*N* = 3)	1.0 mg/kg (*N* = 3)	3.0 mg/kg (*N* = 3)	10 mg/kg (*N* = 3)	Overall (*N* = 12)
Baseline characteristics					
Median age, years (min–max)	78 (63–83)	56 (47–67)	63 (52–63)	63 (62–68)	63 (47–83)
Median ECOG PS (min–max)	1 (1–2)	0 (0–1)	0 (0–1)	0 (0–0)	0 (0–2)
Ongoing maintenance therapy, *n*					
Thalidomide/lenalidomide (Pt. #)	2 (02, 03)	3 (04, 05, 06)	2 (08, 09)	1 (10)	8
Dexamethasone/prednisolone (Pt. #)	2 (02, 03)	2 (05, 06)	2 (08, 09)	1 (12)	7
Cyclophosphamide (Pt. #)	0	1 (05)	0	0	1
Median number of lines of prior antineoplastic therapy^a^ (min–max)	7 (2–8)	6 (5–10)	4 (3–6)	7 (6–11)	6 (2–11)
Patients with prior ASCT, *n* (%)	0 (0%)	2 (67%)	3 (100%)	3 (100%)	8 (67%)
Number of patients (%) with AEs [number of AEs]					
All treatment-emergent AEs					
Grade 1–3	2 (66%) [4]	3 (100%) [3]^b^	1 (33%) [2]	2 (67%) [9]	8 (67%) [18]
Grade 4/5	0	0	0	0	0
Total	2 (67%) [4]	3 (100%) [3]	1 (33%) [2]	2 (67%) [9]	8 (67%) [18]
Possibly, probably, or definitely related AEs					
Grade 1–3	0	0	0	2 (67%) [6]	2 (17%) [6]
Grade 4/5	0	0	0	0	0
Total	0	0	0	2 (67%) [6]	2 (17%) [6]
Possibly, probably, or definitely related AEs by System Organ Class					
MedDRA preferred term					
Grade 1–3	0	0	0	2 (67%) [6]	2 (17%) [6]
Grade 4/5	0	0	0	0	0
Total	0	0	0	2 (67%) [6]	2 (17%) [6]
Gastrointestinal disorders					
Eructation	0	0	0	1 (33%) [1]	1 (8.3%) [1]
Nausea	0	0	0	2 (67%) [2]	2 (17%) [2]
Total	0	0	0	2 (67%) 3]	2 (17%) [3]
Musculoskeletal and connective tissue disorders					
Pain in extremity	0	0	0	1 (33%) [1]	1 (8.3%) [1]
Total	0	0	0	1 (33%) [1]	1 (8.3%) [1]
Respiratory, thoracic and mediastinal disorders					
Dyspnoea	0	0	0	1(33%) [1]	1 (8.3%) [1]
Total	0	0	0	1 (33%) [1]	1 (8.3%) [1]
Vascular disorders					
Flushing	0	0	0	1 (33%) [1]	1 (8.3%) [1]
Total	0	0	0	1 (33%) [1]	1 (8.3%) 1]

*AE* adverse event, *ASCT* autologous stem cell transplantation, *ECOG PS* European Cooperative Oncology Group performance status, *MedDRA* Medical Dictionary for Regulatory Activities, *N* number of patients in treatment group, *n* number of patients, *Pt#* patient identification number, *SD* standard deviation

^a^ASCTs were not included

^b^One patient experienced grade 3 arthralgia

In the 12 patients (*n* = 3/dose) treated with MDX-1097, no dose-limiting toxicities or serious adverse events (AEs) were reported and the maximum-tolerated dose was not reached at the highest dose (10.0 mg/kg). During the study there was a low incidence of AEs, primarily grade 1 or 2 in severity, which resolved without sequelae. The most frequently reported MDX-1097-related AE was nausea (Table 1) at the highest dose level (10.0 mg/kg). One patient experienced a grade 1 infusion reaction with flushing, dyspnoea and nausea after the start of infusion. The infusion was paused and within 30 min the symptoms resolved without treatment and dosing was completed without further issue. A second patient experienced grade 1 AEs on day 2 post infusion with nausea and eructation (resolved on day 15) and pain in extremity (resolved on day 8). No patients experienced a dose-limiting toxicity or discontinued the study due to an AE. There were no dose-related trends or other clinically important safety findings. No indications of treatment-emergent renal impairment were observed and there were no clinical or laboratory parameters, suggesting immune complex formation or serum sickness in any patient treated with MDX-1097. Testing for human anti-chimeric antibody at day 45 post infusion revealed that no antibody response to MDX-1097 was detected in any patients.

**Fig. 1:**
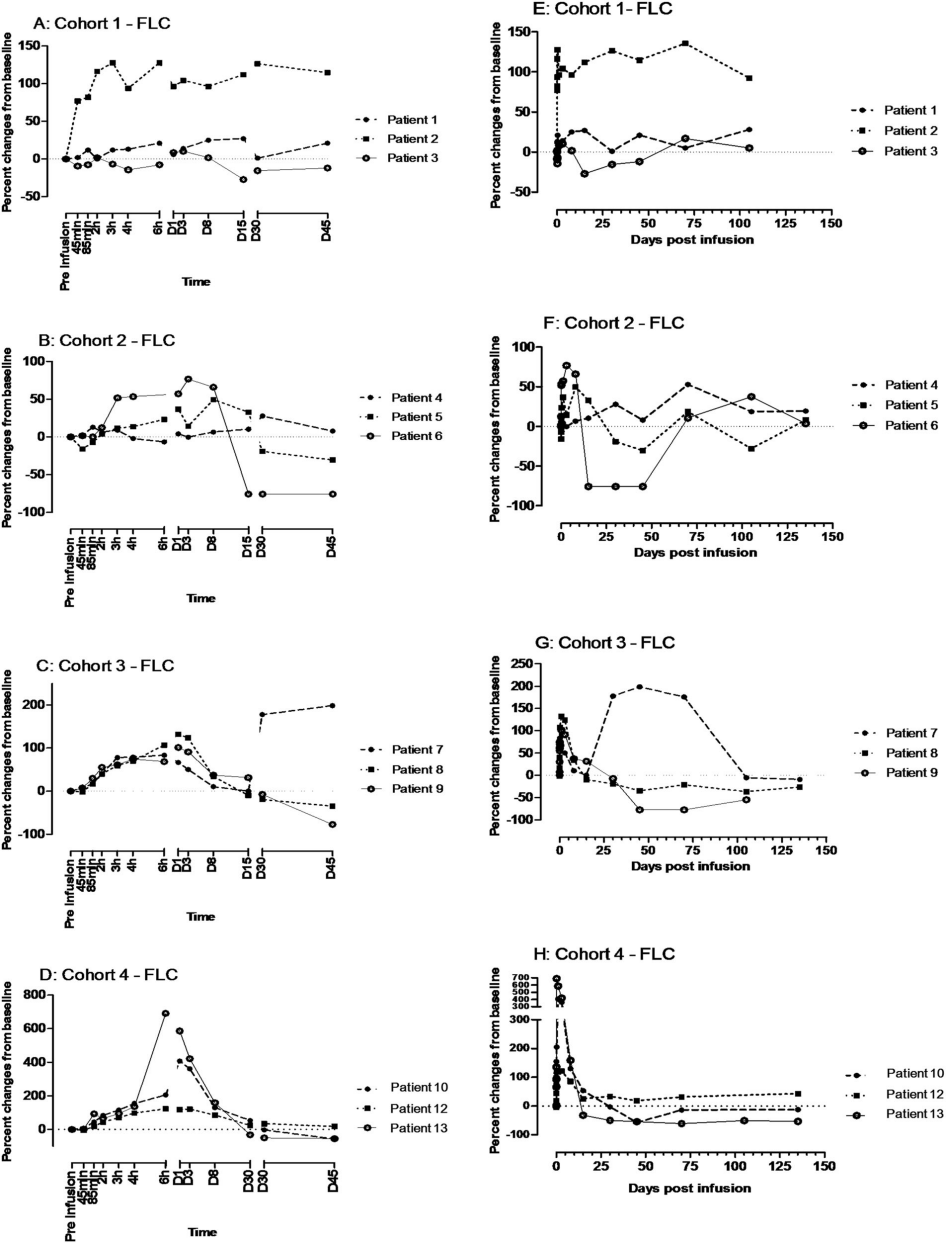
Patient profiles of percent change in serum κ free light chain (κFLC) concentrations from baseline after MDX-1097 intravenous infusion, presented by dose cohort. Cohort 1 (0.3 mg/kg; **a**, **e**), Cohort 2 (1.0 mg/kg; **b**, **f**), Cohort 3 (3.0 mg/kg; **c**, **g**), and Cohort 4 (10 mg/kg; **d**, **h**). Baseline serum concentrations were assessed at −30 min pre-infusion (0) and then post infusion at specified intervals up to day 45 (**a–d**) and during the follow-up phase for a total of 135 days (**e–h**). There was no apparent change in serum λFLC concentration throughout the study and the changes in serum FLC ratios (κ:λ) were consistent with the fluctuations in serum κFLC concentrations. κFLC, kappa free light chain; λFLC, lambda free light chain

We observed an increase in serum κFLC levels following MDX-1097 infusion in all patients, with peak levels reached between days 1 and 15 and then declining to baseline values by day 45 in the majority of patients (Fig. 1). Due to the small cohort size and the variability in baseline serum κFLC levels, there was no clear dose dependency between the serum κFLC increase observed between days 1 and 15 and MDX-1097 concentration. However, the greatest increases were generally observed in the two highest dose groups (3.0 and 10.0 mg/kg; Fig. 1).

Although no conventional disease responses were observed, following a single dose of 3.0 mg/kg in one patient (#8) with non-secretory κ light chain-only restricted MM, we observed an almost complete metabolic response based on a ^18^fluorine-D-glucose (FDG)-positron emission tomography (^18^FDG-PET) scan when compared with baseline data. This patient had been on long-term lenalidomide treatment at the time of both baseline and treatment scans. Before treatment with MDX-1097, the patient had stable extensive skeletal myelomatous disease (Supplementary Fig. 1A), but 30 days after MDX-1097 treatment, repeat scanning demonstrated significant resolution of previously noted FDG-avid lesions apart from an area of disease in the left femur, which exhibited reduced but incomplete resolution (Supplementary Fig. 1B). Importantly, the patient had symptomatic improvement with diminished bone pain and normalisation of kidney function that persisted for 3 months post infusion. In addition, the patient (#13) showed a sustained decrease (>50%) in serum κFLC.

MDX-1097 PK analysis revealed that MDX-1097 mAb clearance increased with increasing dose, suggesting that there was cell-associated antigen clearance (Supplementary Table 2). The MDX-1097 PK profile did not fit the typical “antigen-mediated sink” phenomenon, which is characterised by dose-dependent decreases in antibody clearance as the antigen-mediated clearance mechanisms become saturated at higher antibody concentrations^9^. On the contrary, the antibody clearance increased with increasing dose. MDX-1097 has a lower affinity for soluble antigen compared with the cellular antigen; therefore, preferential binding to KMA due to “avidity” of bivalent binding is likely^7^. In addition, the derived volume of distribution (*V*_*z*_) was consistent with the confinement of MDX-1097 to the blood and extracellular fluid spaces, suggesting that off-target binding of MDX-1097 is unlikely (Supplementary Table 2).

Serum biomarker analyses showed statistically significant, MDX-1097-dose-dependent decreases in serum concentrations of six cytokines that play a significant role in the signalling pathways associated with survival of MM cells in the BM microenvironment (BME) (Supplementary Table 3). The six cytokines that exhibited MDX-1097 dose-related decreases in serum concentration included chemokine (C-X-C motif) ligand 9 (CXCL9), chemokine (C-X-C motif) ligand 10 (CXCL10), macrophage inhibitory factor, hepatocyte growth factor, chemokine (C-C motif) ligand 27, and granulocyte-colony-stimulating factor (G-CSF) (Supplementary Fig. 2), all play a role in B cell trafficking and potential homing from secondary lymphoid organs to the BME^10,11^. No increases in inflammatory cytokines were observed. Although the cytokine data need to be interpreted with caution due to small patient numbers, the observed modulation in the context of MDX-1097 exposure mandates further evaluation.

This study demonstrated that MDX-1097 has a favourable safety profile with no significant drug-related haematologic AEs or SAEs reported. These positive safety findings may be associated with the restriction of KMA expression to κ-type MM cells and occasional tonsillar cells. In contrast, the cell-surface antigen CS1 (SLAMF7, CD319), which is the target for elotuzumab, is highly expressed on myeloma cells, but is also present on normal PCs, natural killer cells, and other immune cells^12^. In the elotuzumab phase I study, low-grade infusion reactions were the most common AEs; however, drug-related SAEs were also observed^13^. Transient decreases in absolute lymphocyte counts resulted from drug-induced increases in the chemokine CXCL10 (IP10); by comparison, MDX-1097 decreased serum levels of this chemokine^13^. Interestingly, in our study antibodies to MDX-1097 were not observed, whereas anti-drug antibodies were relatively common in the elotuzumab phase I study^13^.

Based on this preliminary study a dose level of 10.0 mg/kg was selected for multiple-dose studies based on its favourable safety and PK profile for weekly dosing. A multiple-dose phase 2a study was started with MDX-1097 to assess the safety and efficacy of MDX-1097 in previously treated MM patients with stable measurable disease, but this study was terminated early in order to expedite a phase 2b study with MDX-1097 as a single agent versus MDX-1097 combined with lenalidomide/dexamethasone in patients with relapsed/refractory MM. This decision was based on both the preliminary study and the phase 2a study showing that no AEs were observed in patients on maintenance lenalidomide therapy following dosing of MDX-1097. In addition, our existing data show that lenalidomide increases KMA expression and enhances MDX-1097 activity^7^; hence, there was a compelling rationale for combining MDX-1097 with lenalidomide. The primary objective is to assess overall response rate. Preliminary phase 2b results will be available mid-2019.

## Supplementary information

Supplementary Materials for Preliminary Study on KappaMab in multiple myeloma

Supplementary Figure 1

Supplementary Figure 2
