# Supplementary material for: A preliminary study of the anti-κ myeloma antigen monoclonal antibody KappaMab (MDX-1097) in pretreated patients with κ-restricted multiple myeloma
Source: Blood Cancer J. 2019 Jul 31;9(8):58. doi: 10.1038/s41408-019-0217-5 (PMC6668455; doi:10.1038/s41408-019-0217-5)
Supplement: Supplementary file 1 — Supplementary Materials for Preliminary Study on KappaMab in multiple myeloma [file 41408_2019_217_MOESM1_ESM.docx]

**Supplementary Materials: Preliminary study of the anti-kappa myeloma antigen monoclonal ntibody KappaMab (MDX-1097) in pretreated patients with kappa-restricted multiple myeloma**

**Supplementary Methods**

*Study design*

A 3+3 design was used to investigate safety, dose limiting toxicity, pharmacokinetics (PK), pharmacodynamics, and biologically relevant dose (BRD) of MDX-1097. Drug-target modulation of cell signalling pathways was determined by measuring pro- and anti-inflammatory cytokines, chemokines, and growth factors in serum. The study was approved by the Human Research Ethics Committee at The Alfred Hospital in Melbourne, Australia and was conducted in accordance with the Declaration of Helsinki. Clinical trial registry: [www.anzctr.org.au](http://www.anzctr.org.au); ACTRN12608000336381. Informed consent was gained from all participants.

*Treatment protocol*

The study comprised treatment (Days 1-45), and follow-up (Days 46-135) phases. Thirty patients were to be recruited in 5 MDX-1097 dose cohorts (0.3, 1.0, 3.0, 10.0, and 30.0 mg/kg) with 3 to 6 patients per cohort. After review of the data up to Day 45 for patients in Cohorts 1 to 3 (0.3 mg/kg, 1.0 mg/kg, and 3.0 mg/kg dose cohorts), it was established that MDX-1097 could be detected at all dose levels (up to Day 30), with 0.3 mg/kg the lowest BRD. Therefore, the decision was made to not recruit patients into the highest dose cohort (30.0 mg/kg; Cohort 5). Thus, thirteen patients entered the study, one of whom was found to be ineligible as the myeloma cells expressed only λ light chain. All patients completed the study on Day 45 in accordance with the protocol and protocol amendments.

Dose escalation proceeded in the next higher dose cohort provided that no DLTs (≥grade 3 non-haematological toxicity or grade 4 haematological toxicity using National Cancer Institute Common Toxicity Criteria Version 3.0) occurred within 2 weeks of MDX‑1097 administration. MDX-1097 was infused intravenously over 90 minutes; premedication was not mandated, however, paracetamol, corticosteroids, and antihistamines were allowed for treatment of infusion reactions. Patients were followed up on Days 75, 105, and 135 post infusion for safety.

*Human anti-chimeric antibodies assay*

Blood samples were collected at pre-infusion and Day 45 post infusion for measurement of MDX-1097 human anti-chimeric antibody (HACA) using ELISA. The presence of HACA was measured using a phase-appropriate qualified sandwich ELISA. Briefly, MDX-1097 was immobilised on ELISA plates and the captured antibodies were detected using biotinylated MDX-1097 and ExtrAvidin^®^‑alkaline phosphatase (Sigma, USA) followed by the alkaline phosphatase (AP) substrate. Goat anti-human IgG was used as the positive control for the assay. Day 45 was selected as the HACA post-treatment assessment time point given that patients were immunocompromised, potentially resulting in a delayed immunogenic response. Further, during qualification of the HACA assay, we demonstrated that serum concentrations of MDX-1097 above 0.1 µg/mL abrogated the signal. Therefore, the analysis of HACA was conducted at a later time point post infusion, when MDX-1097 was no longer detected in patients’ serum.

*Pharmacokinetic analyses*

The PK analysis included serum MDX-1097 measurement using a validated ELISA. Briefly, immobilised human kappa Bence Jones protein (Bethyl, Sigma, USA) was used to capture MDX-1097. The captured MDX‑1097 was then detected using an AP-conjugated anti-human IgG (gamma chain specific; Sigma, Cat. No. H4522) followed by colour development with the AP substrate.

*Pharmacodynamic analyses*

Serum FLC levels were quantified using the FreeLite™ assay (The Binding Site Group Ltd, Birmingham, UK). Laboratory evaluation of patients’ serum free light chain (FLC) levels in the presence of MDX-1097confirmed that the antibody does not interfere with nephelometry measurements. However, because the FreeLite™ assay uses a polyclonal anti-κFLC antibody, measurements of serum κFLC in patients treated with MDX-1097 most likely include unbound κFLC and κFLC bound to MDX-1097.

*Biomarker analysis*

Patient serum samples were assessed for biomarkers of MDX-1097 activity after blood samples were collected at -30 minutes before infusion of MDX-1097 and then at 6, 48, 192, and 1,080 hours after infusion. Samples were analysed in triplicate and serum levels of 48 human cytokines, chemokines, and growth factors were assessed. These biomarkers were analysed using the Bio-Plex Pro™ Human Cytokine 21-plex and 27-plex assays (Bio-Rad, Carlsbad, CA, USA; Cat No MF0005KMII, M500KCAF0Y).

*Statistical Analyses*

Multivariate analysis of the biomarker data was performed using R version 3.1.0 (2014‑04‑10) software. Linear mixed effects modelling was completed using lmer (Bates *et al,* 2015). The significance of interactions terms, and interaction means were obtained using the *Phia* package (De Rosario-Martinez, 2015). Multiple test correction was performed using the false discovery rate correction (Benjamini and Hochberg, 1995).

To find significant associations between MDX-1097 dose, time, and cytokine expression levels, we fitted a linear mixed-effects model that modelled cytokine fluorescence expression levels with fixed-effects terms for baseline cytokine fluorescence expression, MDX-1097 dose (with 4 levels: 0.3, 1.0,3.0, and 10 mg/kg), time (treated as factor with 5 levels), and cytokine with 48 levels, plus their interactions, with a random effect associated with patient (12 levels) and plex (2 levels). Plex was used to distinguish between the two cytokine panels/plates. In R notation the statistical model is given as:

$$Val.y\sim Val.x+ Time.y*Dose*Protein+\left( 1 | Plex:Patient \right)$$

Here, Val.y represents cytokine expression at time points defined by time.y (ie, Time.y = 6, 48, 192, and 1,080 hours after infusion). Val.x represents the baseline expression for each cytokine.

**Supplementary Table 1.** Number of lines of prior antineoplastic therapies in patient administered a single dose of MDX-1097

| **Antineoplastic Agent** | **Number of patients*** |
| --- | --- |
| Melphalan | 12 |
| Dexamethasone | 10 |
| Cyclophosphamide | 10 |
| Thalidomide | 10 |
| Prednisolone | 10 |
| Idarubicin | 5 |
| Other clinical trial agent | 5 |
| Lenalidomide | 4 |
| Vincristine | 4 |
| Adriamycin | 3 |
| Dexamethasone | 3 |
| Etoposide | 3 |
| Cisplatin | 3 |
| Bortezomib | 2 |
| Cyclophosphamide/melphalan/dexamethasone | 1 |
| Melphalan/dexamethasone/thalidomide | 1 |
| Melphalan/dexamethasone/prednisolone | 1 |
| Lenalidomide/dexamethasone | 1 |
| Adriamycin/doxorubicin | 1 |
| Clarithromycin | 1 |
| Doxorubicin | 1 |

* Some patients were re-administered an agent in another line of treatment, however each agent is only counted once per patient.

**Supplementary Table 2** Summary of the pharmacokinetic parameters for MDX-1097 following single intravenous doses

|  | Dose of MDX‑1097 | | | |
| --- | --- | --- | --- | --- |
| Parameter | 0.3 mg/kg (N=3) | 1.0 mg/kg (N=3) | 3.0 mg/kg (N=3) | 10 mg/kg (N=3) |
| AUC_0‑tz_ (µg•h/mL) | 803 (23.4) | 3006 (45.3) | 6864 (17.0) | 22994 (15.8) |
| AUC_0‑∞_ (µg•h/mL) | 901 (18.7) | 3079 (46.1) | 6927 (16.9) | 23227 (15.8) |
| C_max_ (µg/mL) | 5.44 (11.5) | 20.6 (24.2) | 69.9 (14.1) | 219 (21.7) |
| t_max_**^*^**(h) | 3.00 (2.00-6.00) | 4.00 (3.00-4.08) | 2.00 (2.00-3.00) | 4.00 (3.00-6.00) |
| AUC_0‑tz_**^†^** (µg•h/mL) | 2678 (23.4) | 3006 (45.3) | 2288 (17.0) | 2299 (15.8) |
| AUC_0‑∞_**^†^** (µg•h/mL) | 3003 (18.7) | 3079 (46.1) | 2309 (16.9) | 2323 (15.8) |
| C_max_**^†^** (µg•h/mL) | 18.1 (11.5) | 20.6 (24.2) | 23.3 (14.1) | 21.9 (21.7) |
| t_½_ (h) | 237 (24.9) | 202 (58.1) | 191 (10.1) | 124 (40.3) |
| CL (mL/min/kg) | 0.00555 (18.7) | 0.00541 (46.1) | 0.00722 (16.9) | 0.00718 (15.8) |
| V_z_ (L/kg) | 0.114 (41.2) | 0.0946 (12.3) | 0.120 (27.3) | 0.0770 (32.5) |

AUC_0-tz)_, area under the concentration versus time curve from time zero to z hours after dose; AUC_0-∞_, area under the concentration versus time curve from time zero to infinity; CL_,_ apparent total body clearance; C_max_, maximum observed concentration; CV, coefficient of variation; N = number of patients studied; t_1/2_, apparent elimination half-life; t_max_, time of maximum observed concentration; V_z_, apparent volume of distribution during terminal phase.

Note: data presented as geometric mean (CV%).

**^*^**Data presented as median (min‑max).

**^†^**Data normalized for dose (mg/kg).

Supplementary Table 3 Statistical significance of decreases in cytokine expression across MDX-1097 doses

| Cytokine | *P* value | Statistical significance |
| --- | --- | --- |
| Chemokine (C-X-C motif) Ligand 9 (CXCL9) | 0.002 | ++^*^ |
| Chemokine (C-X-C motif) Ligand 10 (CXCL10) | 0.031 | +**^†^** |
| Macrophage inhibitory factor (MIF) | 0.031 | + **^†^** |
| Hepatocyte growth factor (HGF) | 0.031 | + **^†^** |
| Chemokine (C-C motif) Ligand 27 (CCL27) | 0.037 | + **^†^** |
| Granulocyte-colony stimulating factor (G-CSF) | 0.037 | +**^†^** |

Note: *P*-values have been multiple-test corrected according to the false discovery rate correction procedure of Benjamini & Hochberg^‡^. Only those cytokines with statistically significant (*P*<0.05) dose-related changes in expression are reported here. For a list of all 48 cytokines analysed in the Bio-Plex Pro™ Human Cytokine 21-plex (Cat No. MF0005KMII and 27-plex (Cat No. M500KCAF0Y) assays please see the product information available at www.bio-rad.com (Bio-Rad, Carlsbad, CA, USA).

^*^ (*P* <0.01)

**^†^**(*P* <0.05)

^‡^ Benjamini, Y. & Hochberg, Y. (1995) Controlling the false discovery rate: a practical and powerful approach to multiple testing. *J R Stat Soc Series B Stat Methodol,* **57,** 289–300.

Supplementary Figure 1 ^18^Fluorine-D-glucose-positron emission tomography (^18^FDG-PET) scans for Patient 8 (who was receiving lenalidomide) prior to MDX-1097 treatment (Panel A) and 30 days after intravenous infusion with 3.0 mg/kg MDX-1097 (Panel B). ^18^FDG-PET scanning was performed encompassing the skull vertex down to the knees on the combined PET/Computed Tomography (CT) scanner and a contemporaneous low-dose, non-contrast CT scan was performed for the purposes of attenuation correction and to provide anatomical correlation. Standard uptake values were not available as the PET scans were not conducted at the study center. In Panel A, multifocal, avid disease is demonstrated within the bone marrow, both within the axial and appendicular skeleton. In the spine, disease is most prominent within the sacrum. In the appendicular skeleton, disease is most pronounced within the proximal femur, where a prosthetic rod is *in situ.* No metabolically active disease is present outside the bones. In Panel B, ^18^FDG-PET scanning was performed as before to assess the response 30 days after MDX-1097 treatment. Uptake in the previously documented multifocal bony abnormalities was largely resolved and indicated a favourable and almost complete metabolic response. Persisting uptake is seen in the medial condyle of the left femur but is less intense than previous scan. Low-grade uptake on the right lower lateral chest wall is intense and may reflect physiological muscular activity. No abnormal uptake is seen in the lungs, liver, or spleen and no nodal uptake is detected.

**Supplementary Figure 2.** Bar plots for MDX-1097 dose effects on cytokine expression levels. Bars represent expression-adjusted means for the 6 cytokines with statistically significant observed interactions (*P*-value <0.05; Table VI) between cytokine expression and MDX-1097 dose. Error bars represent 95% confidence levels. Means are adjusted according to cytokine baseline expression levels and for patient:plate effects. The Y-axis presents the log2 of the mean cytokine fluorescence values (FI). CXCL9 (chemokine (C-X-C motif) ligand 9: HGF, hepatocyte growth factor; MIF, macrophage inhibitory factor, CXCL10 (chemokine (C-X-C motif) ligand 10; CCL27 (chemokine (C-C motif) ligand 27); G-CSF, granulocyte colony-stimulating factor.

**Supplementary References**

Bates, D., Maechler, M., Boker, B. & Walker, S. (2015) Fitting linear mixed-effects models using 1me4. *Journal of Statistical Software,* **67,** 1-48.

Benjamini, Y. & Hochberg, Y. (1995) Controlling the false discovery rate: a practical and powerful approach to multiple testing. *J R Stat Soc Series B Stat Methodol,* **57,** 289–300.

De Rosario-Martinez, H. phia: Post-hoc interaction analysis. R package; 2015. Available at: http://CRAN.R-project.org/package=phia.
