# Supplementary material for: A preliminary study of the anti-κ myeloma antigen monoclonal antibody KappaMab (MDX-1097) in pretreated patients with κ-restricted multiple myeloma
Source: Blood Cancer J. 2019 Jul 31;9(8):58. doi: 10.1038/s41408-019-0217-5 (PMC6668455; doi:10.1038/s41408-019-0217-5)
Supplement: Supplementary file 2 — Supplementary Figure 1 [file 41408_2019_217_MOESM2_ESM.docx]

**Supplementary Figure 1**


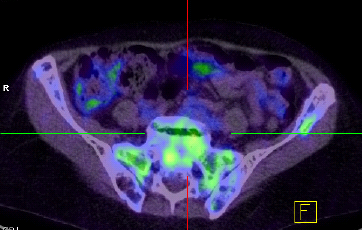

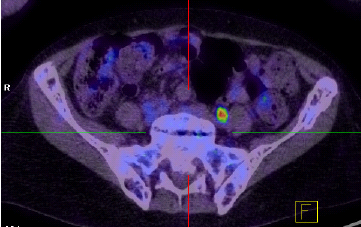

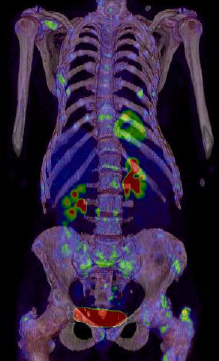

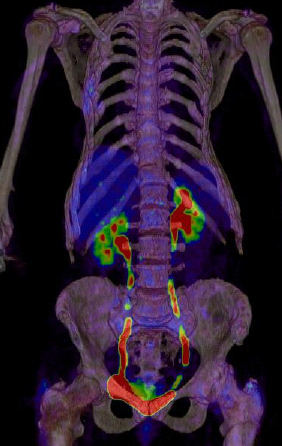


(B) PET scan taken on July 2009, 30 days post infusion of MDX-1097 (patient continued lenalidomide)

(A) PET scan taken on February 2009 prior to enrolment in study (patient was treated with lenalidomide)
