# Supplementary figures and images for: A preliminary study of the anti-κ myeloma antigen monoclonal antibody KappaMab (MDX-1097) in pretreated patients with κ-restricted multiple myeloma
Source: Blood Cancer J. 2019 Jul 31;9(8):58. doi: 10.1038/s41408-019-0217-5 (PMC6668455; doi:10.1038/s41408-019-0217-5)

**Supplementary Figure 2**


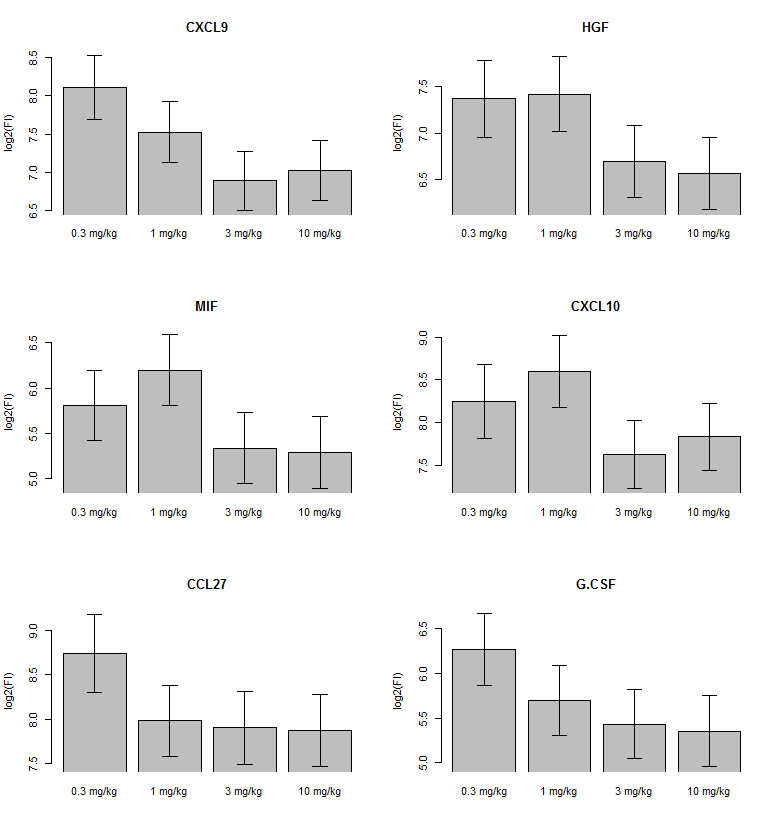

Supplement: Supplementary file 3 — Supplementary Figure 2 [file 41408_2019_217_MOESM3_ESM.docx]
